# A Comparative Evaluation of the Shear Bond Strength of Three Different Hybrid Tooth-Colored Restorative Materials to Dentin: An In-Vitro Study

**DOI:** 10.7759/cureus.60123

**Published:** 2024-05-11

**Authors:** Kishan Agarwal, Leeza Bharati, Shreya Agarwal, Varnika Yadav, Jaydip Marvaniya, Ritwik Shyamal

**Affiliations:** 1 Department of Conservative Dentistry and Endodontics, Awadh Dental College and Hospital, Jamsedhpur, IND; 2 Department of Conservative Dentistry and Endodontics, Private Practice Clinic, Guwahati, IND; 3 Department of Conservative Dentistry and Endodontics, Deen Dayal Upadhyay Hospital, Delhi, IND; 4 Department of Conservative Dentistry and Endodontics, Career Dental College, Lucknow, IND; 5 Department of Conservative Dentistry and Endodontics, Private Practice Clinic, Contai, IND

**Keywords:** zirconomer, cention n, vitremer, shear, glass ionomer

## Abstract

Introduction: Silver amalgam, glass ionomer, resin-modified glass ionomer, compomers, light polymerized hybrid composite resin, and hybrid glass ionomer are among the most frequent restorative materials used as cavity-based or post-endodontics. Thus, to meet the needs of both patients and dentists, Cention N reimagines the traditional filling by integrating bulk placement, ion release, and durability into a dual-curing, aesthetically pleasing solution. Hoewver, we do not have enough information from studies comparing this hybrid restorative material's shear bond strengths to dentin to draw any firm conclusions. Cention N, zirconomer, and Vitremer are three hybrid tooth-colored restorative materials that were evaluated for their shear bond strength to dentin. This research aimed to compare and evaluate these materials.

Methodology: The purpose of this research was to use a universal Instron machine to measure the shear bond stress of three distinct hybrid tooth-colored restorative materials in relation to dentin. The research samples consisted of 45 extracted lower first premolars from humans. The teeth were then assigned into three groups of 15 samples each according to different color acrylic resin blocks, namely, group A (pink acrylic blocks), which had Cention in cement; group B (white acrylic blocks), which has zirconomer cement; and group C (violet acrylic blocks), which had Vitremer cement.

Results: There was no statistically significant difference between the three groups and the normal distribution, as shown by the negligible values in the tests involving the three groups. Put simply, each of the three categories exhibits data that follows a normal distribution. This allows for further data analysis to be conducted using the parametric test of significance.

Conclusion: The shear bond strength of hybrid glass ionomer restorative materials has to be further investigated in both laboratory and living organism settings.

## Introduction

One of nature's greatest wonders is the human tooth. On the other hand, its ability to regenerate is somewhat restricted. This calls for the use of a restorative substance to substitute the tooth's structure [1]. Tooth decay, cracks, old fillings, and endodontic therapy might weaken the structure of an endodontically treated tooth [2]. The last stage in a successful root canal treatment is to restore the tooth with a permanent, definitive, post-endodontic restoration. This is because teeth that have had root canals are more likely to fracture [3].

After severe dental caries removal or a tooth fracture, a core or foundation repair is often necessary [4]. It is recommended that a core replacement be performed when the coronal portion of the tooth is lost over 50% [5]. Because bulk fracture is one of the primary causes of restorative failure [6], it is still difficult to find the right posterior restorative material to use in stress-bearing locations. When choosing a core material, strength is an important consideration among several. For optimal performance and therapeutic outcomes, it is best to use core materials that are more robust to withstand deformation and fracture, distribute stress more evenly, and lower the likelihood of failure [7].

Compression, tension, or shear stress [8] may be generated inside a restored tooth as a result of any force applied to it, due to the complicated distribution of stresses along the tooth and restoration interface. The shear bond strength illustrates the adhesive strength of materials at the tooth and restoration interface, which is important because mastication causes indentation, which is essentially linked to shearing [9]. One way to define a material's resilience to structural failure is by looking at its shear strength, which is defined as its resistance to yielding under shear stress and shear loads [10]. Silver amalgam, glass ionomer, resin-modified glass ionomer, compomers, light polymerized hybrid composite resin, and hybrid glass ionomer are among the most frequent restorative materials used as cavity-based or post-endodontics [11]. Core build-up processes have made use of the majority of these materials' features [12].

Because of its physical and chemical characteristics, including its anti-cariogenicity, biocompatibility with pulp, and physiochemical bonding to dentin and enamel, glass ionomer cement (GIC) was introduced to dentistry by Wilson and Kent in 1972 [13]. Traditional GIC has several drawbacks, including being brittle, weak, tough, and having a low resistance to wear. A hybrid material called resin-modified GIC (RMGIC) was created by adding a resin component to the original GIC, which further enhanced its physical and mechanical qualities [14]. In response to the market need for longer-lasting materials, scientists have created a novel material that enhances the properties of glass ionomers by including zirconia filler particles. Utilizing zirconia particles, SHOFU Inc. (Japan) created a new generation of GICs (zirconomer) with improved compressive and flexure strengths, reduced occlusal wear, and a quick setting response. The manufacturer claims it has amalgam's strength and endurance with glass ionomer's protective properties without mercury's dangers [15].

In 1990, not long after RMGICs were introduced, a new class of dental materials called compomers was offered. They promised to combine the advantages of composites (the “comp” in their name) with glass ionomers (the “omer”). [16] The “alkaline” filler used in Cention N makes it possible for the compound to release ions that neutralize acid. Direct restorations made of Cention N, a simple filling material that matches tooth color, are available. Light curing is an optional component of the self-curing process. Thus, the Centurion N satisfies the needs of both patients and dentists by reimagining the traditional filling with its dual-curing, aesthetically pleasing design that combines bulk placement, ion release, and endurance. The literature lacks sufficient data regarding comparisons of shear bond strengths for this hybrid restorative material to dentin. This study was undertaken to compare the in-vitro shear bond strengths of three recently introduced hybrid tooth-colored restorative materials, namely, Cention N (Ivoclar Vivadent), zirconomer (Shofu Dental, Japan), and Vitremer (3M ESPE, St. Paul, MN).

## Materials and methods

This study was conducted in the Department of Conservative Dentistry and Endodontics, Saraswati Dental College and Hospital, Lucknow. Using a universal Instron machine to measure their shear bond stress, the researchers hoped to compare and contrast three distinct hybrid tooth-colored restorative materials' capacity to adhere to dentin. The research samples consisted of 45 extracted lower first premolars from humans. The teeth were debrided by immersing them in 2.5% NaOCl for 24 hours using an ultrasonic scaler. After that, they were placed in distilled water and left at room temperature until the experiment began. This research used extracted lower first premolar teeth from humans ranging in age from 18 to 25 years. The objective is to evaluate the shear bond strength of various dental materials, including Cention N, zirconomer, and Vitremer, both individually and in combination. This involves assessing the adhesive properties of each material and their compatibility with one another. Specifically, the study aims to determine the shear bond strength of Cention N, zirconomer, and Vitremer independently, as well as the bonding strength when Cention N is paired with zirconomer, zirconomer with Vitremer, and Cention N with Vitremer. This comprehensive evaluation will provide insights into the efficacy and potential synergistic effects of these dental materials when used in combination, aiding in the selection and optimization of restorative procedures in dentistry.

Preoperative radiographs were taken with radiovisiography (RVG) and were screened, and then teeth were excluded if any of the following were noted such as the presence of caries, abnormal anatomical structure, external or internal resorption, visible cracks, fractured teeth, and previously restored teeth. The shear bond strength assessment conducted in the study serves as a crucial parameter for evaluating the efficacy of different dental restorative materials in adhering to dentin. By measuring the shear bond strength, researchers can determine the ability of these materials to withstand forces exerted during mastication and other oral functions. This information is essential for clinicians in selecting the most appropriate restorative material for various clinical scenarios, considering factors such as longevity, durability, and resistance to occlusal forces.

**Figure 1:**
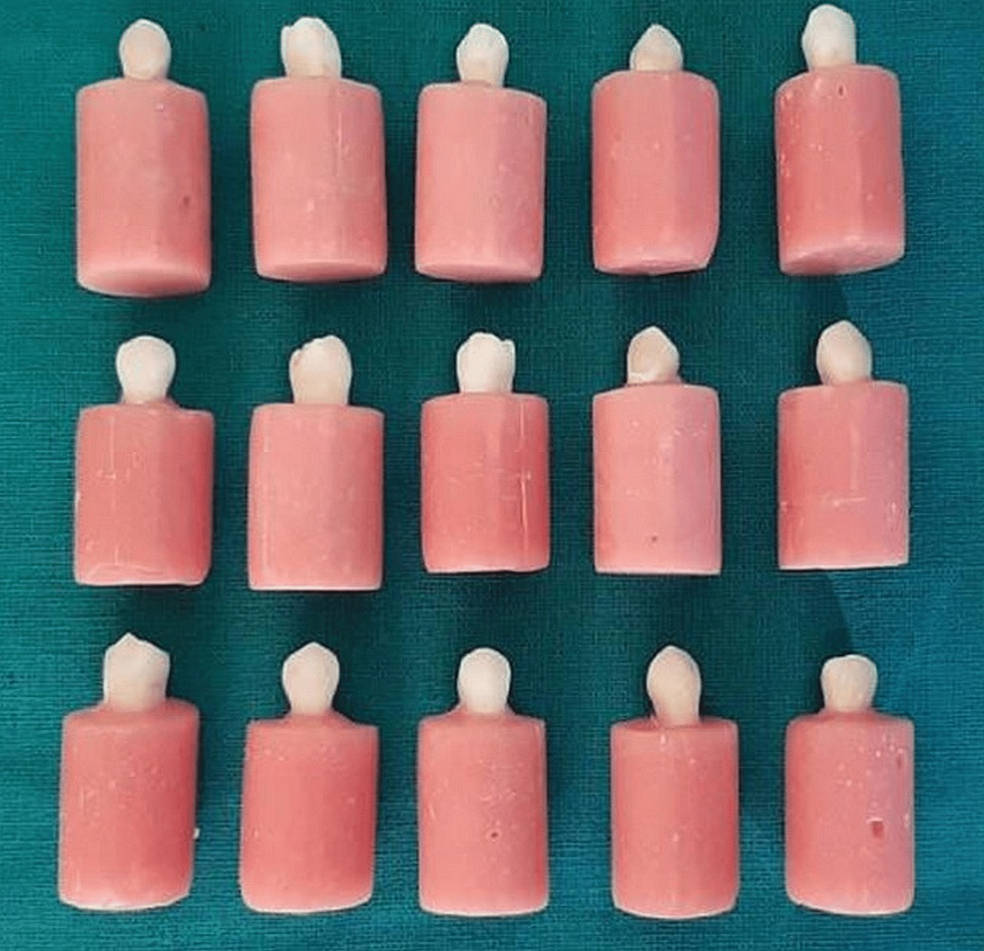
Samples used for the study

The coronal section of the crowns was sliced perpendicular to the tooth's long axis, 1 mm above the dentino-enamel junction, using a low-speed diamond disc (Isomet 2000 Precision saw; Buehler, Lake Bluff, IL) with a generous amount of water spray. Afterward, a 400-grit aluminum oxide abrasive paper was utilized to achieve a smooth dentin surface. All 45 samples were embedded in acrylic resin blocks of three distinct colors (pink, clear white, and purple) in order to distinguish them from one another. The embedding was done up to the level of the cementoenamel junction (CEJ), with the crown section exposed and parallel to the base. The samples were submerged in distilled water at ambient temperature. The study utilized preformed silicon tubes with a height of 5 mm and a diameter of 2.5 mm. These tubes were coated with a non-reactive lubricant, specifically petroleum jelly, on their inner walls. The purpose of this study was to fill the test material in all three groups of occlusal dentin samples. The samples were standardized using these negative forms. The cross-sectional area of the specimen and the test material was placed in direct contact with the dentin surface while ensuring that it did not touch the enamel. Great caution was exercised to prevent the formation of voids, gaps, or any entrapment of air (Figure 1).

The teeth were then assigned into three groups of 15 samples each according to different color acrylic resin blocks, namely, group A (pink acrylic blocks), which had Cention N in cement; group B (white acrylic blocks), which had zirconomer cement; and group C (violet acrylic blocks), which had Vitremer cement.

Thirteen samples were left to air dry in the first group. Each sample's occlusal dentin was etched with 37% orthophosphoric acid for 15 seconds before a 10-second water rinse. Using cotton pellets, samples were dried after washing and blotting. Light curing the occlusal surface for 20 seconds was done with one layer of Coltene Whaledent bonding agent. Someone placed a silicon mold on an occlusal surface. A ratio of 4.6:1 was utilized on a mixing pad, meaning that one scoop of Cention N (Ivoclar Vivadent, Schaan, Liechtenstein) powder was used for every drop of liquid. Following the manufacturer's directions, it was worked into the dentin and then transferred to the mold using a cement carrier. We used a condenser to thoroughly compress the repair, and then we cured it under a light for 20 seconds. In group B, 15 samples were left to air dry. Before being blotted dry using cotton pellets, the occlusal surface of each sample was conditioned with 20% polyacrylic acid for 10 seconds. Based on the instructions provided by the manufacturer, zirconia-reinforced GIC (Zirconomer, Shofu Dental, Tokyo, Japan) material was prepared using a 2:1 powder-to-liquid ratio. Using an agate spatula, the mixture was mixed on a paper pad. With the help of a cement carrier, the mold was restored and condensed well to eliminate voids.

In group C, the remaining 15 samples were air-dried. The occlusal surface was treated by applying primer for 30 seconds and air dry. Light curing was done for 20 seconds. Mixing of Vitremer (3M ESPE, Maplewood, MN) was done according to the standard manufacturer protocol of a powder:liquid ratio of 2.5:1 by weight on a mixing pad with an agate spatula for three minutes. Cement was carried to the mold, which was placed on the occlusal surface with the help of a cement carrier. The restoration was well condensed with a condenser to eliminate voids. Finally, it was cured by a light cure for 40 seconds.

**Figure 2:**
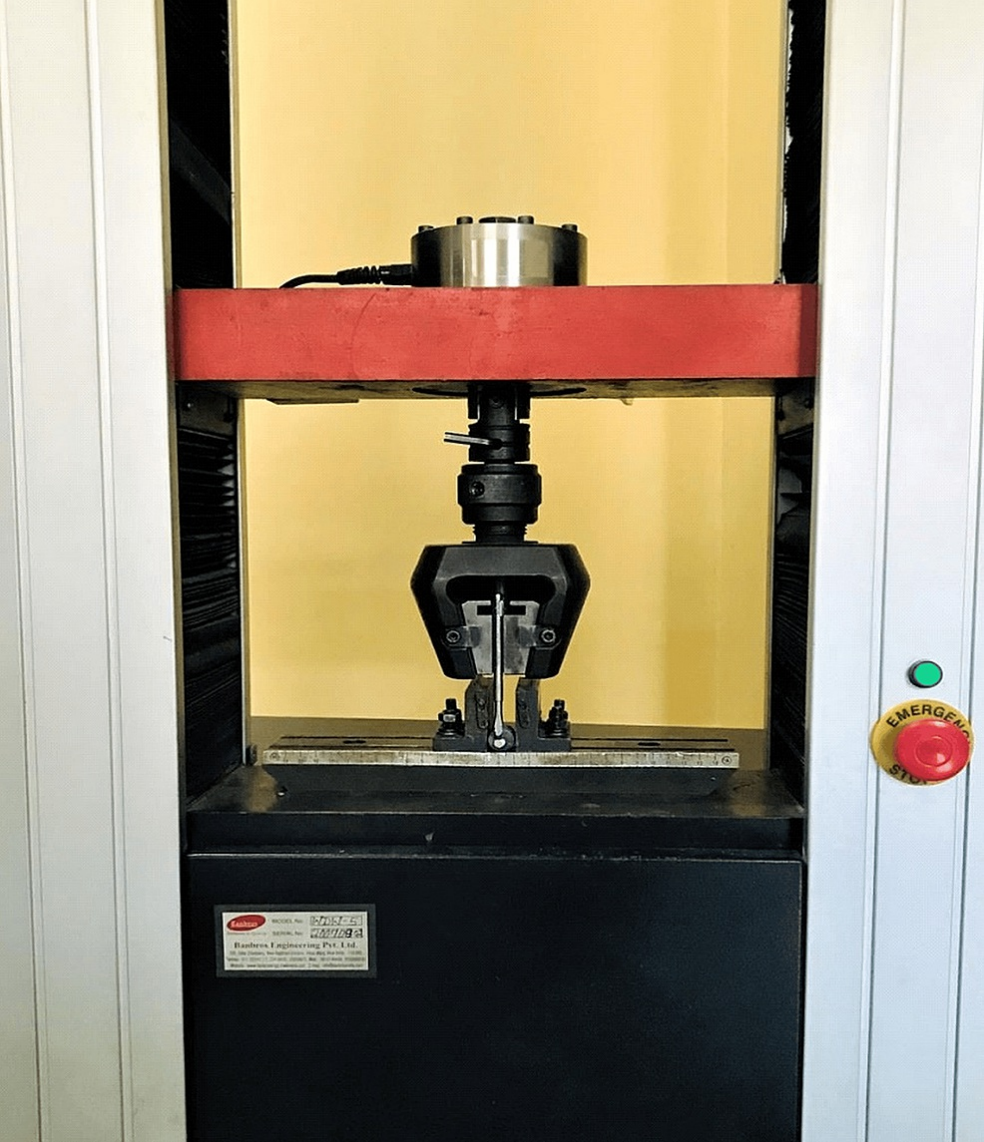
Universal testing machine for material testing

**Figure 3:**
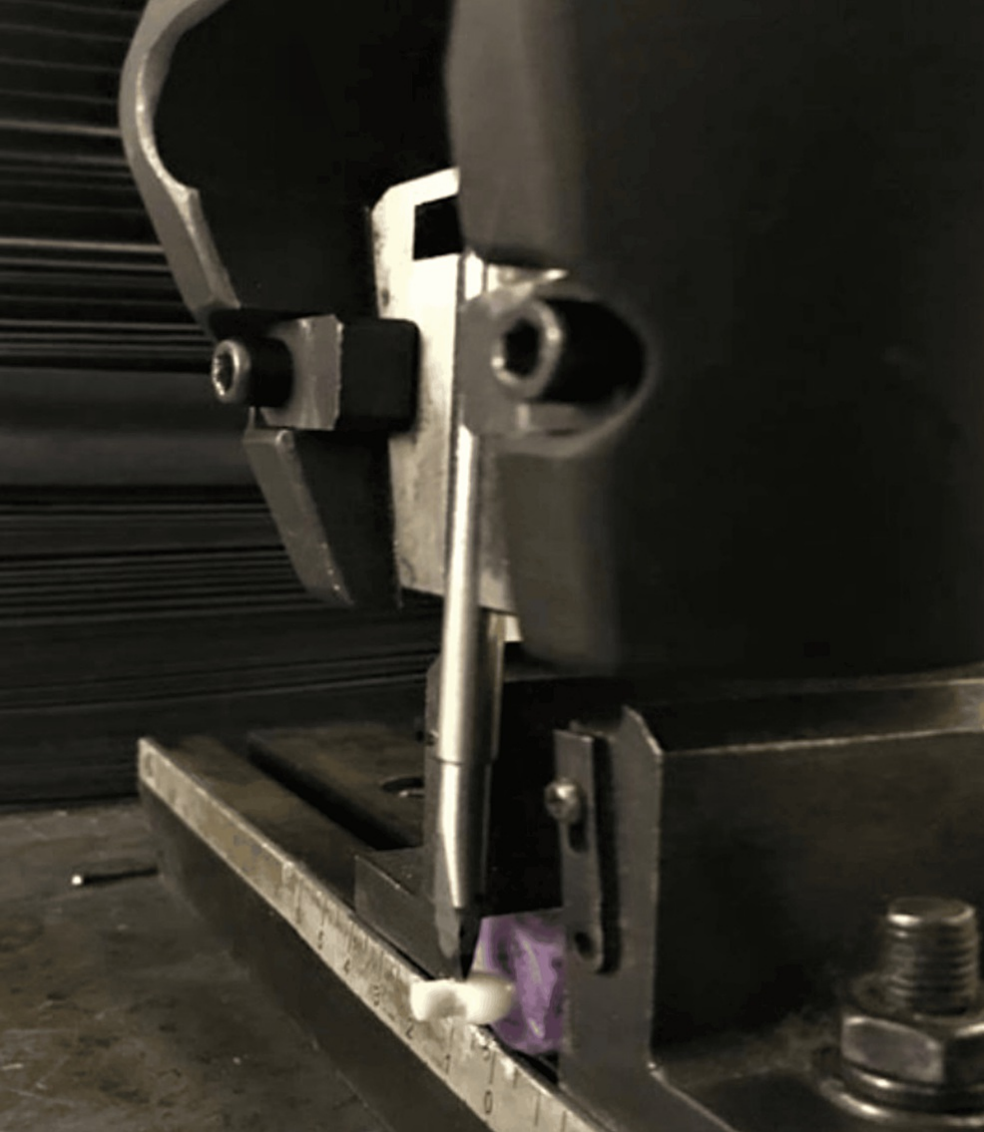
Test showing plunger positioned at the dentin restoration interface

The samples were left in distilled water at 37 degrees Celsius for one day. Once the dentin surface was exposed, the molds were carefully dismantled using a blade until only the cylindrical test material was left attached perpendicularly. In order to drive a metal plunger, all of the specimens were placed in a metal mold within the universal Instron machine (model WDW-5. Banbros Industries, Taiwan). At right angles to the dentin contact point, this plunger made contact with the cylindrical test material (Figures 2, 3).

The ANOVA test was used to examine the data, and the t-test was employed to determine significance based on the p-value. For statistical significance, a p-value of less than 0.05 was used.

## Results

The three categories of restorative materials are described in depth. It shows that, for every group, there are 15 samples. Additionally, it demonstrates that all groups have the same total number of cases (15 in each) and that neither group is missing any cases (Table 1).

**Table 1:** Case processing summary for the normality distribution analysis of the three groups Group A (pink acrylic blocks) - Cention in cement, group B (white acrylic blocks) - zirconomer cement, and group C (violet acrylic blocks) - Vitremer cement

Group	Cases					
	Valid		Missing		Total	
	N	Percent	N	Percent	N	Percent
Group A	15	100.00%	0	0.00%	15	100.00%
Group B	15	100.00%	0	0.00%	15	100.00%
Group C	15	100.00%	0	0.00%	15	100.00%

The specifics of the three groups' normalcy distribution are shown in Table 2. Two tests, the Kolmogorov-Smirnov test and the Shapiro-Wilk test, were conducted to determine if the shear bond strengths in the three groups followed a normal distribution. The results of these tests are shown in Table 2.

**Table 2:** Tests of normality distribution for the three groups Group A (pink acrylic blocks) - Cention in cement, group B (white acrylic blocks) - zirconomer cement, and group C (violet acrylic blocks) - Vitremer cement Kolmogorov-Smirnov and Shapiro-Wilk test used

Group	Kolmogorov-Smirnov			Shapiro-Wilk		
	Statistic	df	Significance	Statistic	df	Significance
Group A	0.145	15	0.200	0.968	15	0.821
Group B	0.107	15	0.200	0.959	15	0.674
Group C	0.146	15	0.200	0.971	15	0.872

Due to the fact that none of the two tests involving the three groups produced statistically significant results, we may conclude that the three groups do not deviate significantly from normal distribution. That is to say, it informs us that the data in all three categories follow a normal distribution, which means we may go on to the parametric test of significance for further analysis.

The individual group shear bond strengths expressed in megapascals (MPa) are been described in Table 3.

**Table 3:** Actual shear bond strength in the three groups

Shear Bond Strength in Megapascals (MPa)	Group A	Group B	Group C
Minimum	13.3	3.8	4.81
Maximum	28.3	10.67	17.83

Table 4 displays the results of the ANOVA that compared the shear bond strengths of the three categories of restorative material. There is a very significant difference in the shear bond strength of the three groups of restorative materials, namely, groups A, B, and C. The p-value is 0.000, which is less than 0.05.

**Table 4:** ANOVA or analysis of variance showing the p-value after comparison of the shear bond strengths of the three categories of restorative material

Variable	Sum of Squares				
		df	Mean Square	F	Sig.
Between Groups	1227.45	2	613.522	51.243	0.001
Within Groups	502.862	42	11.973		
Total	1729.91	44			

## Discussion

Mechanical and physical features, handling characteristics, and the mix of materials are among the many elements that determine the overall effectiveness of dental restorations. The material's capacity to adhere to tooth structures and eradicate microleakage is another crucial success element. Consequently, measuring the shear bond strength was the primary objective of this research.

Three hybrid tooth-colored restorative materials Cention N, zirconomer, and Vitremer were tested for their shear binding strength to dentin in this research. Due to the challenges of evaluating the functional and mechanical properties of dental materials in restored teeth under vivo conditions and the limitations of clinical trials in estimating these properties, the study was conducted in vitro. This allowed for the possibility of evaluating the mechanical properties of both the restored teeth and the materials used for restoration [17]. Due to the high failure rate, endodontically treated teeth provide unique obstacles for dental restoration. As a result, several restorative solutions have been developed to address these tooth's vulnerabilities [18].

In order to rebuild a severely decaying tooth, a core or foundation restoration is used to repair the majority of the tooth's coronal section to its optimum anatomical shape. This is done prior to placing the full coverage crown. In theory, it ought to provide the patient with a functional restoration that lasts a long time. Due to the fact that the core becomes an essential component of the tooth's structure. Although the quantity of retained tooth structure (which is responsible for the appropriate ferrule effect) is the primary determinant of the long-term clinical effectiveness of an indirect restoration, the core build-up material also plays an essential impact. In an ideal world, the material used to construct the core would have superior mechanical qualities that would allow it to withstand any forces applied during operation, distribute those forces evenly, and lessen the likelihood of bond failures.

Composites modified with zirconium particles, resin-modified glass ionomers (RMGIC), grocers (a matrix of organic and inorganic polymers), and compomers (resin-based materials changed by adding polyacid) are new kinds of hybrid materials which are extensively employed in dentistry. Resin composite materials' contraction pressures on enamel and dentin need a minimum bond strength of 17 to 20 MP a, according to the postulation. The primary criterion for material selection is its maneuverability, with mechanical qualities and manipulation factors receiving adequate attention.

The inorganic fillers include barium aluminum silicate, ytterbium trifluoride, an iso-filler, calcium barium aluminum fluorosilicate, and calcium fluorosilicate glass fillers are found in Cention N. The particle size of these fillers ranges from 0.1 μm to 35 μm. Stronger bonds are achieved when silanes attached to filler particles form a chemical link between the matrix and the glass surface. Thanks to the exclusive utilization of cross-linking methacrylate monomers together with a reliable and effective self-cure initiator, Cention N displays an exceptionally high density of polymer networks and degree of polymerization throughout the whole depth of the repair. The need for a more durable material has led to the development of a new material that adds zirconia filler particles to the glass ionomer composition.

In the present in-vitro study, 45 extracted permanent mandibular first premolar teeth were selected, as this sample size gave a significant outcome statistically. The premolar teeth chosen were extracted for orthodontic purposes. Criteria selected for inclusion were, single-rooted teeth, caries, and lesion-free teeth having normal anatomic form and structure. Following the recommendation of Strawn et al. [19], which states that storage solutions may modify the dentin substrate, which can impact bond strength investigations, the teeth that were chosen for cleaning were placed in ultra-pure water after being scaled with an ultrasonic scaler. In order to control factors for comparison testing, this research followed the proposals made by Rueggeberg [20] and Causton [21] to do bond strength testing after sectioning the tooth either 1 mm above the pulp horns or 1 mm below the dentino-enamel junction.

As far as the author is aware, there is a dearth of research on the shear bond strength of zirconomer, Cention N, and Vitremer hybrid GIC, and no studies that directly compare their shear bond strengths. This research stands out since it compares the shear bond strengths of all three materials. Thus, more studies evaluating the shear bond strength of hybrid glass ionomer restorative materials are needed, both in laboratory settings and in living organisms.

Some of the study limitations are the use of extracted human premolars may not fully represent the complex oral environment, which includes factors such as saliva flow, occlusal forces, and temperature variations, all of which could affect the bonding properties of the materials differently than in vitro conditions. The use of extracted lower first premolars from humans ranging in age from 18 to 25 years may introduce bias due to the variability in dentin composition and characteristics among individuals of different ages. The study did not assess the long-term performance or durability of the restorative materials, which is essential for evaluating their clinical applicability and longevity. Therefore, future research should aim to address these limitations by employing larger sample sizes, utilizing more clinically relevant models, and evaluating comprehensive properties of the materials over extended periods to provide more evidence regarding their effectiveness and suitability for clinical use.

## Conclusions

Within the confines of the study limitations, it can be inferred that the newer compomer materials demonstrate superior bonding potential to dentin compared to modifications of GIC. Specifically, Cention N exhibits better shear bonds than zirconomer cement and resin-modified GIC. Nonetheless, these conclusions necessitate further clinical evaluation to validate the findings. Additional in vivo research is essential to corroborate and expand upon the outcomes of this study.
